# Survey of magnetic resonance imaging availability in West Africa

**DOI:** 10.11604/pamj.2018.30.240.14000

**Published:** 2018-07-31

**Authors:** Godwin Inalegwu Ogbole, Adekunle Olakunle Adeyomoye, Augustina Badu-Peprah, Yaw Mensah, Donald Amasike Nzeh

**Affiliations:** 1 Departments of Radiology, University College Hospital Ibadan, Ibadan, Nigeria; 2 Lagos University Teaching Hospital Idiaraba, Idiaraba, Nigeria; 3 Komfo Anokye Teaching Hospital Kumasi, Kumasi, Ghana; 4 Korle Bu Teaching Hospital Accra, Accra, Ghana; 5 University of Ilorin Teaching Hospital Ilorin, Nigeria

**Keywords:** MRI, West Africa, survey, utilization, partnership

## Abstract

**Introduction:**

The availability and utilization of MRI units across sub-Saharan Africa countries remain poor and its distribution is largely unknown. A cross-sectional survey was conducted to determine the distribution and utilization of MRI facilities across the West African sub-region.

**Methods:**

An interview and online search survey was conducted from September 2015 to September 2016, to determine the MRI facilities (Government/Public and Private) available in the West African sub-region. In Nigeria and Ghana, face-to-face interviews were conducted while for other West African countries, telephone interviews with radiologists and other health professionals as well as a Google online search were conducted to ascertain the distribution of the MRI facilities in the region. The number of MRI units in West Africa per million population was calculated and compared with other parts of the world from available published data.

**Results:**

Eighty-four MRI units serve a combined population of 372,551,411 in the West African sub-region at the time of this report. Nigeria accounted for more than two-thirds (58 (69%)) of the available units. Of these, 45 (77.6%) of the units were low-field strength systems. Ghana's 14 MRI units were fairly equally distributed between the private (57%) and the public sectors (43%). Ghana with 0.48 units/million population had the highest number of MRI units/ million population followed by Nigeria with 0.30 units/million population.

**Conclusion:**

Though there is an increase in the number of available MRI units in the West African sub region in the last decade, the numbers remain appallingly small for the population. Infrastructural and maintenance limitations constitute a major impediment to the use of high filed systems in the region. There may be need for greater cooperation between public and private enterprises for future improvement of MRI utilization in the region.

## Introduction

Despite the emergence of Magnetic Resonance Imaging (MRI) over four decades ago in 1974, developing countries in Africa still have limited access to this clinically beneficial and revolutionary imaging modality. Its utilization in West Africa (comprising 16 countries from Nigeria in the east to Senegal in the west, with an estimated population of more than 350 million people) remains low compared to developed nations of the world [1]. MRI remains one of the most wildly used imaging modalities in developed countries, growing exponentially in improvements yearly with the use of higher field strength magnets. In most developing countries, the utilization of MRI is limited due to enormous acquisition costs, lack of infrastructure and the expertise required for maintaining and running the systems [2-4]. The limited personnel with knowledge of MRI physics and clinical applications remain a major predicament of the developing nations. In recent years, many developing countries have undergone economic transformation with higher living standards, growth of the ageing population, increased healthcare awareness and access to advanced health services although the health indices remain poor [5, 6]. The changing healthcare landscape provides a market potential for MRI in West Africa, if the challenges of infrastructure, health service reform and lack of properly trained personnel are addressed [5, 6]. There is a critical need for the development of training programs and deployment of more resources, for MRI facilities to become more affordable in the developing world. This is because, it would be improper, to charge patients for MRI examination at the same cost, as in developed countries. Creating improved access to advanced medical imaging with MRI in the developing world, would be crucial to the improvement its healthcare provision systems. Therefore, a good understanding of the current state of facilities may provide the basis for planning and development of strategies that would address the challenges and tap into the opportunities that may become available in the near future. A cross-sectional survey of the MRI facilities in West Africa was conducted to determine the distribution and utilization of MRI facilities across the sub-region.

## Methods

A one-year survey using both interview and online search was conducted, from September 2015 to September 2016, to determine the MRI facilities (Government/Public and Private) available in the West African sub-region comprising Benin, Burkina Faso, Cape Verde, Ivory Coast, Gambia, Ghana, Guinea, Guinea-Bissau, Liberia, Mali, Mauritania, Niger, Nigeria, Senegal, Sierra Leone and Togo. Hospitals and diagnostic centers in Nigeria were visited for oral interviews and inspection of facilities. For other West African countries, telephone or face-to-face interviews were conducted with radiologists and other health professionals to ascertain the composition of the MRI facility present in their locality. In addition, there was review of available electronic literature to establish reported MRI facilities in West African private and public institutions. The number of MRI machines in West Africa per million population was calculated and compared with other parts of the world from available published data.

## Results

As at September 2016 there were 84 MRI units in the West African sub-region with Nigeria having 58 (69%) accounting for more than two-thirds of the available functional units in the region (Table 1). The number of MRI units in Nigeria was fairly well distributed across all its geo-political zones. However, all the units were found to be located in the urban areas and with Lagos state in the Southwest; the most densely populated city of the country accounting for the highest number of MRI scanners [14 (25%)]. Nearly two-thirds of the MRI equipment in Nigeria, were found also to be located within the private health sector (63%) while the remainder (37%) were located in the public health sector. Forty-five (77.6%) permanent magnet (low-field) units were identified in the West African region (<0.3 Tesla). The remaining 13 (22.4%) units were superconducting magnets (High-field) with 1.5 Tesla field strength. There were no 3Tesla units in the entire region, nonetheless, of the few high-field strength units available, almost three-quarters (73%) were located in the private health system. The 84 MRI machines in West Africa served a combined population of more than 350 million. Ghana, with a population of nearly 30 million, had only 14 MRI machines. Each of the remaining West African countries had at most three MRI machines or none at all (Table 2). Ghana's 14 MRI units were fairly equally distributed between the private (57%) and the public sectors (43%). Comparatively, Ghana had the highest number of MRI units per million population in West Africa, with 0.48 units / million population and Nigeria, coming a close second with only 0.30 units/million population. In North Africa, Libya (5.16) had the highest number of MRI units per million population followed by Tunisia (2.00), Egypt (2.00) and Morocco (0.36) respectively. South Africa had a 0.24 MRI unit per million populations against some other countries in the Southern African sub-region like Namibia and Zimbabwe that had 0.87 and 0.28 units per million respectively (Table 2) [7]. Globally, it is estimated that there are about 36,000 MRI machines in the world with 2,500 being produced yearly. Japan had the highest ratio of MRI units per million population with 51.67, followed closely by the United States of America with 38.96 units/million. France had 12.59, Canada 9.48, Saudi Arabia 0.97, Mexico 2.25, Israel 4.21 and Qatar had 9.22 MRI units per million population (Table 3) [8].

**Table 1:** Distribution of magnetic resonance imaging machines in Nigeria

State/Region	MRI Units	Government (High Field/Low Field)	Private (High Field /Low Field)
Lagos	14	0/2	5/7
Abuja	10	2/3	3/2
Port Harcourt	6	0/1	0/5
Kano	3	0/0	0/3
Ibadan	2	0/1	0/1
Ilorin	2	0/1	0/1
Zaria	2	0/1	0/1
Enugu	2	0/0	0/2
Jos	2	0/1	0/1
Delta	2	1/0	0/1
Yenagoa	2	0/2	0/0
Maiduguri	1	0/1	0/0
Ile-Ife	1	0/1	0/0
Nnewi	1	0/1	0/0
Bauchi	1	0/1	0/0
Sokoto	1	0/1	0/0
Kaduna	1	0/1	0/0
Gombe	1	0/0	0/1
Umuahia	1	0/0	0/1
Ondo	1	0/0	0/1
Yola	1	0/0	0/1
Calabar	1	0/0	0/1
Total	58	21(3/18)	37(8/29)

**Table 2:** Distribution of MRI Units in the West African Sub-Region (per million)

Country	Population (2017)	MRI Units	(Approx. Per Million Population)	GDP	Human Development Index
*Nigeria	191,835,936	58	0.30	2,929.525	0.514
*Ghana	28,656,723	14	0.48	1,384.354	0.579
Mauritania	4,266,448	3	0.77	1,197.121	0.506
*Ivory Coast	23,815,886	2	0.08	1425.056	0.462
Senegal	16,054,275	2	0.14	945.863	0.466
*Guinea	13,290,659	1	0.07	519.173	0.411
Burkina Faso	19,173,322	1	0.06	644.502	0.402
Togo	7,691,915	1	0.15	586.301	0.484
Gambia	2,120,418	1	0.54	435.452	0.441
Cape Verde	533,468	1	2.00	3,057.916	0.646
Benin Republic	11,458,611	0	0.00	814.360	0.480
Mali	18,689,966	0	0.00	844.274	0.419
Liberia	4,730,437	0	0.00	478.681	0.430
Niger	21,563,607	0	0.00	412.797	0.348
Sierra Leone	6,732,899	0	0.00	635.892	0.413
Guinea Bissau	1,932,871	NA	NA	624.671	0.420
**Total**		**83**			

* Information originating from by the current survey

**Table 3:** Distribution of MRI Units in selected countries globally (per million population)

Country	MRI units (approx. /million)	GDP
*Japan (2014)	51.67	32,477
*USA (2014)	38.96	56,116
*South Korea	26.47	27,222
*Spain (2014)	15.30	25,832
*France	12.59	36,206
*Portugal	9.90	19,222
Turkey (2014)	9.81	9,126
*New Zealand	9.62	37,808
*Canada	9.48	43,249
*Qatar	9.22	73,653
*Saudi Arabia	0.97	20,482
Mexico (2014)	2.25	9,008
Serbia	6.20	5,235
Libya	5.16	4,642
*Israel	4.21	35,728
*Uruguay	2.94	15,574
Tunisia	2.00	3,873
Morocco	0.36	3,004
Egypt	2.00	3,615
South Africa	2.90	5,724

* Countries with GDP >10,000

## Discussion

The present survey is the first comprehensive analysis of MRI capacity and utilization in the West African sub-region and provides new insight to the degree of availability of advanced health care facilities in the sub-region using MRI as a diagnostic tool. Generally, many countries on the African continent and other low and middle-income countries (LMIC) have relatively minimal contribution of MRI to their diagnostic armamentarium. A number of the West African countries have no MRI machine or not more than 1 MRI unit per million of their population. On the basis of regional assessment of MRI utilization in Africa, countries in the Northern sub-region of Africa appear to have a relatively good MRI availability and presence, and show better MRI utilization per million compared to other parts of the continent [1, 5, 9-11]. MRI utilization in Africa is quite low when compared to the leading industrial nations in the world e.g Japan, United States, France and Spain. This may be attributed to a better economic status and financial standing in these more technologically advanced countries. In addition, the existence of first grade health care policies that support the partnership between government and private institutions, in the provision of healthcare and health insurance services, may play a role [12, 13]. Participation of the private sector in health investment in Africa, appears to be improving with sub-Saharan African countries, recording a steady pattern of economic growth in recent years. The African continent has become one of the most attractive investment destinations in the world. Foreign direct investment (FDI) in the region has increased fivefold compared to its 2000 level [14] (Table 3). In recent times, there has been a perceptible increase of investment in private health care provision, as well as standalone diagnostic facilities across the West African sub-region. Although this has had some positive impact on the value of advanced imaging with MRI in the health sector, services still remain fragmented, poorly regulated with great variability in delivery of quality outcomes [12, 13, 15-17]. This pattern is clearly observable in the Nigerian healthcare environment, where the greatest impact of MRI utilization, is found in the private sector and predominantly in the urban centers. These facilities serve only a limited population of users who are able to afford the services, as access is only by out of pocket payments, with little or no health insurance coverage for use of such services.

It appears that the general economic potential of a nation, to a large extent, has an effect on the distribution of MRI facilities. Countries with a relatively good GDP per capita and a commendable (third or fourth quartile) Human development index (HDI) also have a relatively good MRI unit per million population value. West African countries have the lowest GDP per capita values compared to major countries in other sub-regions in Africa. Cape Verde ($3,057.916) and Nigeria ($2,929) however, have GDPs above $2000 compared to most of the other countries, having a GDP of less than a thousand dollars. This survey observed that, usually countries with Human Development Index (HDI) above 0.500 have at least 3 MRI units and above, with Cape Verde being the only exception. The HDI is used as a measure of economic development and welfare. It uses three main criteria of economic development (life expectancy, education and income levels) to create an overall score of between 0 and 1. Zero (0) representing a very low level and 1 indicates a high level of economic development. It is used to determine whether a country is regarded as developed, developing or underdeveloped. That is the reason why there is still a low utilization of MRI in the Western African sub-region as most countries have HDI of < 0.5. Some other reasons have been given for the poor availability of this useful diagnostic tool for healthcare. Amongst these is the predicament, prompted by inequities in workforce distribution and “brain drain” of skilled and educated workforce, out of the sub-region for “greener pastures” in other continents of the world. Thus, the expected availability of human resources in most African nations for effective public health interventions is lacking, particularly in remote rural areas [18]. Most of the rural towns in West African countries do not have good hospital infrastructure that could accommodate an MRI unit, as part of its health management facility or investigative tool. Therefore, there is the tendency for seekers of quality healthcare to look elsewhere and may have to travel to the urban cities, to get access to these investigative health facilities. Additionally, the cost of an MRI examination, which is usually a function of the cost of the machine; cost of expertise required to operate it amongst others, can be a dilemma especially for the teeming poor population in sub Saharan Africa. The citizens of these nations live below the poverty index and there is generally lack of access to good health insurance policy/plan to cover the costs of the examination.

Health financing in West Africa is characterized by low government investment in health, lack of comprehensive health financing policies and strategic plans, extensive out-of-pocket payments, lack of social safety nets to protect the poor, weak financial management, inefficient resource use, and weak mechanisms for coordinating partner support in many of the countries in the sub-region. People are familiar with the problems the health sector in Sub-Saharan Africa faces: poor infrastructure, a shortage of healthcare workers, a lack of capacity in the existing workforce and substandard facilities. These problems, coupled with inadequate public sector spending on health, have pushed the private sector to the forefront in the development of the African health sector. Most of the developed countries in the world make use of high magnetic field MRI machines which allow for a greater availability of more advanced imaging capability such as: fat saturation techniques, magnetic resonance angiography (MRA), functional magnetic resonance imaging (fMRI) and magnetic resonance spectroscopy (MRS). In comparison, the low magnetic field MRI machines that are more widely available in West African countries, have only basic imaging functions which are restricted to fewer techniques and limited scope in diagnosis. Regardless of this drawback however, these low magnetic field machines still play a major and invaluable role in the management of neurological and musculo-skeletal disease conditions in West Africa. The high field MRI units are not easily acquired by the government institutions in these countries due to prohibitive costs of purchase and maintenance, which are usually far above the normal budgetary provisions. Also, lack of appropriate and stable electricity infrastructure remains a huge challenge and therefore poses daunting limitations for the use of high field MRI machines that require cryogens in these countries.

## Conclusion

Though there is an increase in the number of available MRI units in the West African sub-region in the last decade. The numbers remain appallingly small for the population. Infrastructural and maintenance limitations constitute a major impediment to the use of high-field systems in the region. There may be need for greater cooperation between public and private enterprises for future improvement of MRI utilization to reverse the situation in the region. Equipment vendors should play a more active role in the deployment of MRI Units through partnership within both the private and public health sectors for the enrichment of advance investigational imaging services in the West African region.

### What is known about this topic

- There is lack of local infrastructure in African countries such as no provision for supply of liquid helium and unreliable electrical power service;
- Satellite networking infrastructure providing symmetrical 384-kbit/s Internet service has been previously implemented to achieve practical intercontinental transport of DICOM image MRI data via the Internet.

### What this study adds

- Low-field MRI systems predominate in the West African sub-region and are mostly located in urban centers and owned by private institutions;
- There is a great disparity of MRI availability in the west African sub-region compared with other regions of the world;
- There are signs of minimal increase of MRI facilities over the last 10 years, the region remains underserved and however public and private partnership with local institutions may bridge the gap to improve availability of MRI in the region.

## Competing interests

The authors declare no competing interests.
